# Teachers' Participation in Decision-Making, Professional Growth, Appraisal, and Behavioral Intentions in the Promotion System Reform in Chinese Universities

**DOI:** 10.3389/fpsyg.2022.932324

**Published:** 2022-06-30

**Authors:** Wangxin Peng, Subadrah Madhawa Nair

**Affiliations:** ^1^Faculty of Education and Liberal Studies, City University Malaysia, Petaling Jaya, Malaysia; ^2^School of Foreign Languages, Hanshan Normal University, Chaozhou, China

**Keywords:** promotion system, reform, teacher empowerment, Chinese university teachers, behavioral intentions, decision making, professional growth

## Abstract

The promotion system in Chinese universities has been undergoing a reform since 2017. This study employed an online survey validated by confirmatory factor analysis with 372 Chinese teachers to investigate the extent to which they were empowered by the two practices of participation in decision-making and professional growth in the reform and level of their appraisal of and behavioral intentions toward the new promotion system. Structural equation modeling was used to measure how the two empowerment practices influenced the teachers' appraisal of and behavioral intentions toward the new system. The findings suggest that the Chinese teachers had low participation in decision-making and medium institutional support for professional growth, relatively low nonmonetary cost-benefit appraisal and medium practicality and fairness appraisal of the new system, and relatively high behavioral intentions to increase efforts according to the new system. Besides, participation in decision-making had a significantly direct effect on practicality and nonmonetary cost-benefit appraisal. Professional growth had a significantly direct effect on practicality, fairness, and nonmonetary cost-benefit appraisal and behavioral intentions. Nonmonetary cost-benefit appraisal had a significantly direct effect on behavioral intentions. The implications are that, in promotion system reforms, the two empowerment strategies of shared decision-making and professional growth can help establish a new promotion system with high nonmonetary cost-benefits for teachers and raise teachers' behavioral intentions to develop and pursue promotion. They can also contribute to the formulation of a new promotion system that effectively evaluates individual teacher's achievements according to the characteristics of the specific university, teacher type, and discipline. This study had the limitations of using convenience sampling, collecting cross-sectional data through self-administered questionnaires, and reporting only teachers' side of the story. Therefore, it is recommended that future studies target both teachers and administrators, employ a mixed-method design, collect quantitative data through random sampling, and take a longitudinal view.

## Introduction

The promotion system in Chinese universities has undergone significant changes since 2017. Before 2012, among the 1,145 universities offering degree programs in China, only 123 were entitled to grant associate professorships and 175 were entitled to grant professorships (China's Ministry of Education, 2012), which means that the other 847 universities had to rely on provincial departments of education for granting academic titles. The old title promotion system before 2017 was not without its drawbacks. Compared with teachers of science, it takes a longer process for teachers of arts to carry out research and publish papers (Peng, 2019). However, instead of acknowledging the difference between disciplines, the old promotion system in some Chinese provinces imposed the same criteria on teachers of different disciplines for promotion (Peng, 2019), which might have produced negative influences on teachers' academic development. Therefore, Peng (2019) recommended that, for foreign language teachers with lower research productivity than teachers of other arts disciplines, universities should consider formulating supportive evaluation rules to encourage these teachers to utilize their foreign language competence and engage in English medium publication (Peng, 2019).

In an effort to delegate power and streamline administration, China's Ministry of Education and Ministry of Human Resources and Social Security (2017) issued a policy that stipulated that individual universities have the autonomy in appraising teachers' research, teaching, and social services achievements and granting academic titles. Individual universities could have taken this opportunity to draw up a new title promotion system that scientifically evaluates teachers based on the characteristics of the specific disciplines to which they belong and motivates them to develop professionally by referring to the evaluation rules.

However, recent studies have reported that the new promotion system in some universities is not effectively differentiating between different disciplines or teacher types (Xing and Yuan, 2021; Zhang and Liu, 2022). While some universities have formulated different evaluation rules for the three subject areas of natural science, social science, and others (including art, physical education, and foreign language teaching), the judges appointed are not representative of the various disciplines, which is unfair to teachers of some disciplines (Fang, 2022). What is more, it is highly likely that teachers of different disciplines may have to compete for an annual quota of promotion, which will place teachers of some disciplines at a disadvantage (Liu, 2018; Fang, 2022; Niu and Zhang, 2022). Similarly, while most universities have formulated different evaluation rules for the three teacher types of research-focused, teaching-research-focused, and teaching-focused, the rules are not distinguishing enough (Han, 2021; Zhang and Liu, 2022).

In fact, if universities aim to set up a promotion system that fairly appraises teachers of different disciplines and teacher types and effectively promotes faculty's academic development, it is necessary that they consider the differences between disciplines and teacher types and adjust the research requirements for different disciplines and teacher types according to their research productivity (Peng, 2019; Han, 2021; Niu and Zhang, 2022; Zhang and Liu, 2022). This is also true for teachers' development in teaching and social services and for different university types, e.g., vocation-oriented universities and research-intensive universities. An important measure to effectively address the differences is empowering ordinary teachers by involving them in the formulation of the new promotion system, understanding their difficulties and needs for professional development, and providing them with necessary support.

However, while the policy (China's Ministry of Education and Ministry of Human Resources and Social Security, 2017) required that individual universities should seek faculty's opinions when drawing up the new promotion system, it did not specify to what extent universities should accept faculty's opinions, make corresponding adjustments to the evaluation rules, and offer corresponding support for professional development. Therefore, it is possible that some universities may not have seriously consulted their faculty members in the formulation of the new system or offer necessary support for their pursuit of promotion. After all, previous studies have shown that national and school-level educational decisions are often made in a top-down approach and Chinese teachers are expected to comply (Lai and Lo, 2007; Lee et al., 2011; Meng and Sun, 2019; Lei and Xu, 2022). It is also possible that this lack of shared decision-making and institutional support may have resulted in the above drawbacks of the new promotion system in terms of practicality and fairness reported by scholars (e.g., Liu, 2018; Han, 2021; Xing and Yuan, 2021; Fang, 2022; Niu and Zhang, 2022; Zhang and Liu, 2022). However, these possibilities are just speculations and need to be validated by empirical evidence.

Therefore, it is imperative to conduct this study to quantitatively measure (1) to what extent Chinese teachers are empowered by the two practices of participation in decision-making and professional growth in the promotion system reform; (2) how they appraise the new promotion system; (3) how they intend to act under the new system; (4) how the two empowerment practices of shared decision-making and professional growth influence their appraisal of and behavioral intentions toward the new system; and (5) how their appraisal of the new system influences their behavioral intentions.

The following six research questions were formulated to achieve the research objectives:

1. What are Chinese teachers' participation in decision-making and institutional support for professional growth in the promotion system reform?
2. What are Chinese teachers' practicality appraisal, fairness appraisal, and nonmonetary cost-benefit appraisal of the new promotion system?
3. What are Chinese teachers' behavioral intentions toward the new promotion system?
4. To what extent does participation in decision-making directly affect practicality appraisal, fairness appraisal, nonmonetary cost-benefit appraisal, and behavioral intentions?
5. To what extent does institutional support for professional growth directly affect practicality appraisal, fairness appraisal, nonmonetary cost-benefit appraisal, and behavioral intentions?
6. To what extent do practicality appraisal, fairness appraisal, and nonmonetary cost-benefit appraisal directly affect behavioral intentions?

## Literature Review

### Participation in Decision-Making and Professional Growth

Empowerment is viewed as a multifaceted social process where the individuals gain control over their own lives and exercise influence on community governance (Rappaport, 1987; Zimmerman and Rappaport, 1988). It is a construct that involves both the individuals developing competencies and an environment offering opportunities for the individuals to develop and display the competencies (Katz, 1984; Rappaport, 1987; Zimmerman and Rappaport, 1988). Empowered individuals believe that they have the competencies to not only act on a situation but also improve it (Short, 1994). In the past few decades, empowerment has become a popular research topic in education. Overall, teacher empowerment has been conceptualized from three perspectives.

First, some scholars examine teacher empowerment as a multifaceted process (e.g., Short and Rinehart, 1992; Short, 1994; Klecker and Loadman, 1998; Bogler, 2005; Lee et al., 2011; Avidov-Ungar and Arviv-Elyashiv, 2018; Tindowen, 2019; Ahrari et al., 2021). Teacher empowerment is defined as a process where teachers develop the competencies to take charge of their own development and address their own problems (Maeroff, 1988; Short, 1994). In the School Participant Empowerment Scale developed by Short and Rinehart (1992), there are six dimensions of teacher empowerment, namely, involvement in decision-making, professional growth, status, self-efficacy, autonomy, and impact. Involvement in decision-making refers to the involvement of teachers in school decisions that have direct influences on their work, e.g., financial matters, curriculum, and teacher selection (Short, 1994). Professional growth refers to teachers' perceptions that they enjoy opportunities offered by the institution to learn, expand their skills, and develop professionally (Short, 1994). Status refers to teachers' perceptions that they have professional respect from their colleagues and the public (Short, 1994). Self-efficacy refers to teachers' perceptions that they are competent in helping students learn (Short, 1994). Autonomy refers to teachers' sense of freedom to control certain aspects of their work (Short, 1994). Impact refers to teachers' perceptions that they can influence their colleagues and students (Short, 1994). It can be seen that involvement in decision-making and professional growth are the two dimensions that are closely related to the context of a promotion system reform since a promotion system reform inevitably involves the formulation of new evaluation rules and the provision of development opportunities to meet the new requirements.

There are also many scholars investigating teacher empowerment from the socio-structural perspective (e.g., Vecchio et al., 2010; Sagnak, 2012; Lee and Nie, 2013, 2014) and the psychological perspective (e.g., Thomas and Velthouse, 1990; Spreitzer, 1995; Moye et al., 2005; Lee and Nie, 2013, 2014; Meng and Sun, 2019; Lei and Xu, 2022). While participation in decision-making is also included in their conceptions, professional growth is neglected. For example, Sagnak (2012) measured school principals' leadership empowering behavior in four dimensions, namely, improving work meaningfulness, enhancing involvement in decision-making, expressing confidence, and granting autonomy. Enhancing involvement in decision-making, which roughly corresponds to participation in decision-making, includes the sample item of “My principal makes many decisions together with us” (Sagnak, 2012, p. 1638). Spreitzer (1995) measured teachers' psychological empowerment in four dimensions, namely, meaning, competence, self-determination, and impact. Impact, which roughly corresponds to participation in decision-making, refers to teachers' perceptions that they can influence the school's strategic, managerial, and operating decisions (Ashforth, 1989). The reason why professional growth is a unique dimension for scholars taking the multifaceted process perspective is probably that they tend to view teacher empowerment as a process where teachers develop competencies and the institution offers opportunities for teachers to develop and display the competencies (Katz, 1984; Rappaport, 1987; Maeroff, 1988; Zimmerman and Rappaport, 1988; Short, 1994).

In fact, participation in decision-making and professional growth are inseparable empowerment strategies in the discourse of teacher development. According to previous studies (OECD, 2009; Tay et al., 2021), some of the significant barriers in teachers' attempts at professional growth include a lack of suitable content and school support and the nomination of all teachers for the same type of compulsory courses. As proposed by Bainer and Wright (1998) and Kelly and Williamson (2002), teachers should be empowered with the freedom to make choices about their professional development so that they can effectively engage in professional development. Eun (2008) also argued that teachers' needs and goals should be accurately assessed to improve the effectiveness of professional development activities. Involving teachers in the planning of professional development activities and reflecting teachers' needs and goals in the activities will be an advisable measure to increase the chance of achieving good outcomes (Tay et al., 2021). Meanwhile, the school should recognize that teachers' desire for professional growth is beneficial to the school (Bogler and Somech, 2004) and that professional development should be integrated into the school calendar and culture (Kelly and Williamson, 2002). Therefore, when the school makes plans for teachers' professional development activities, it is critical to involve teachers in decision-making, understand their needs for professional growth, and offer corresponding support.

Similarly, when the school reforms its promotion system that is closely related to teachers' professional development, it is also important to consider if the two empowerment strategies of participation in decision-making and professional growth can increase the chance of achieving good outcomes, i.e., whether the reform will promote teachers' engagement in development and pursuit of promotion. To the best of our knowledge, however, there have been insufficient studies that combine the two dimensions of participation in decision-making and professional growth in the investigation of how to increase the chance of achieving good outcomes in promotion system reforms in higher education.

Nevertheless, there have been quite many studies that broadly measure teachers' participation in decision-making of teacher issues and teachers' professional growth in primary and secondary schools. For example, Bogler (2005) reported that for the 983 secondary school teachers surveyed in Israel, they had a low actual involvement in managerial decision-making (e.g., teacher evaluation). Cheng (2008) reported that for the 335 secondary school teachers surveyed in Hong Kong, China, their actual participation level in decision-making of managerial issues (e.g., human resource management) was much lower than their desired level. Sarafidou and Chatziioannidis's (2013) survey of 143 primary school teachers in Greece concluded that there was a gap between their intended and actual participation levels in all the three domains of student, teacher, and managerial issues. Their actual involvement level in decision-making concerning student issues was higher than teacher issues (e.g., teacher behavior and professional development) and managerial issues (Sarafidou and Chatziioannidis, 2013). Regarding teachers' perceived level of professional growth, in most cases it is moderately higher than participation in decision-making, as shown in the Chinese primary and secondary educational context (Lee et al., 2011), the Israeli primary and secondary educational context (Avidov-Ungar and Arviv-Elyashiv, 2018), and the Pilipino Catholic higher educational context (Tindowen, 2019).

### Behavioral Intentions Toward Reforms

Teachers' receptivity to an educational reform is an essential factor that determines whether the reform will be successfully implemented (Waugh and Godfrey, 1993). Receptivity usually involves the three dimensions of attitude, overall feelings, and behavioral intentions (Waugh and Godfrey, 1993). Attitude and overall feelings are the two aspects concerning teachers' general opinions about the reform, i.e., whether they think it is worthwhile and whether they applaud or dislike it (Waugh and Godfrey, 1993). In contrast, the dimension of behavioral intentions is closely related to action. Behavioral intentions are defined as teachers' direct intentions to behave in the reform, i.e., whether they will support or oppose it (Waugh and Godfrey, 1993).

For successful implementation of an educational reform, it is important to identify the variables that significantly influence teachers' behavioral intentions toward the reform so that policymakers can tailor the reform accordingly (Waugh and Godfrey, 1993). So far, studies have mainly focused on teachers' behavioral intentions toward curricular reforms in primary and secondary education. In the Chinese context, three variables have been identified as having significant influences on teachers' behavioral intentions to implement a proposed curricular reform, namely, teachers' nonmonetary cost-benefit appraisal of the reform, teachers' practicality appraisal of the reform, and school support. For example, Lee (2000) surveyed 1,687 primary school teachers in an environmental educational reform in Hong Kong, China, and found that teachers' behavioral intentions to implement the proposed reform were significantly predicted by their nonmonetary cost-benefit and practicality appraisal of the system and perceived support. In particular, nonmonetary cost-benefit appraisal accounted for the largest percentage of the variance of behavioral intentions (Lee, 2000). Yin et al. (2011) surveyed 1,366 primary and secondary school teachers in a curricular reform in southwestern China and found that teachers' behavioral intentions to implement the proposed reform were significantly predicted by their nonmonetary cost-benefit and practicality appraisal of the system and perceived support. Nonmonetary cost-benefit appraisal accounted for the largest percentage of the variance of behavioral intentions (Yin et al., 2011).

Regarding the effects of teachers' participation in decision-making and professional growth on teachers' behavioral intentions toward educational reforms, studies were rather limited in number and also focused on curricular reforms in primary and secondary education. For example, in the western context, Waugh and Godfrey (1993) surveyed 480 Australian secondary school teachers and identified that their behavioral intentions to implement the new curriculum was significantly but weakly predicted by their participation in decision-making of the reform. However, in the Chinese context, participation in decision-making seems like a less effective strategy to improve teachers' behavioral intentions toward curricular reforms. Lee et al. (2011) surveyed 1,646 primary and secondary school teachers in a national curricular reform in China and discovered that teachers' participation in decision-making and professional growth did not significantly predict their behavioral intentions to carry out the reform. Instead, teachers' nonmonetary cost-benefit and practicality appraisal of the reform and institutional support significantly predicted their behavioral intentions (Lee et al., 2011).

Nevertheless, Lee et al. (2011) took practicality appraisal and nonmonetary cost-benefit appraisal merely as independent variables without considering them also as dependent variables. That is to say, the influences of participation in decision-making, professional growth, and institutional support on the teachers' practicality appraisal and nonmonetary cost-benefit appraisal of the curricular reform were unknown. In fact, when examining a promotion system reform, it is also essential to know how participation in decision-making and institutional support for professional growth influence teachers' practicality and nonmonetary cost-benefit appraisal of the reform if policymakers aim to improve the practicality and nonmonetary cost-benefits of the new promotion system.

### Participation in Decision-Making, Professional Growth, Appraisal, and Behavioral Intentions: An Integrated Model

As shown by the above review, the existing teacher empowerment and receptivity literature tended to focus on teachers and curricular reforms in primary and secondary education. In contrast, there have been insufficient studies that combine the conceptions of teacher empowerment and teacher receptivity to investigate teachers' promotion and development issues in higher education. Therefore, in this study, we investigated teacher empowerment through participation in decision-making and professional growth in the reform of the promotion system in Chinese universities and its influences on teachers' appraisal of the new system and behavioral intentions to increase efforts according to the new system.

**Figure 1:**
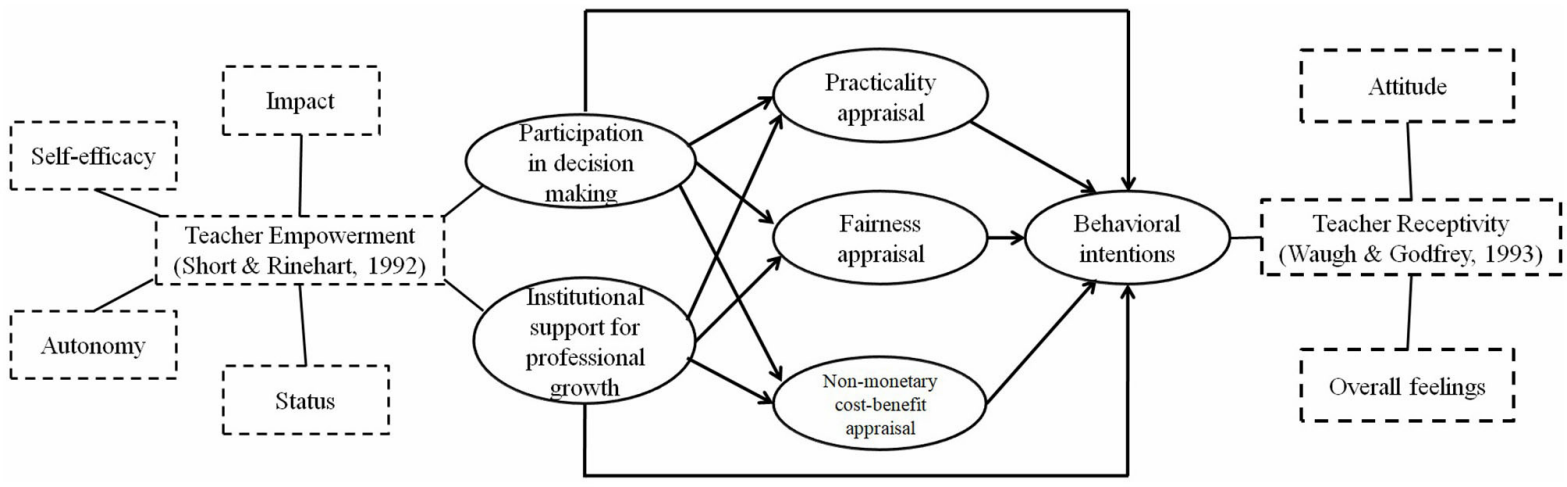
Theoretical framework of the study.

Based on the teacher empowerment literature and teacher receptivity literature, we built the following theoretical model (see Figure 1). Teacher empowerment contains the two dimensions that are closely related to promotion system reforms, namely, participation in decision-making and professional growth (Short and Rinehart, 1992). Participation in decision-making refers to the involvement of teachers in the formulation of the new title promotion system. Professional growth refers to teachers' perceptions that they enjoy opportunities offered by the university to pursue development in research, teaching, and social services.

Although teacher receptivity toward educational reforms involves the three dimensions of attitude, overall feelings, and behavioral intentions (Waugh and Godfrey, 1993), this study focused on behavioral intentions in that we are concerned about how the new promotion system affects teachers' actual engagement in research, teaching, and social services. Behavioral intentions are defined as teachers' direct intentions to behave under the new promotion system, i.e., whether they will increase their efforts in research, teaching, and social services according to the new evaluation rules. As empirical evidence suggested that teacher's behavioral intentions toward educational reforms are affected by institutional support, nonmonetary cost-benefit and practicality appraisal of the reforms, and participation in decision-making (e.g., Waugh and Godfrey, 1993; Lee, 2000; Lee et al., 2011; Yin et al., 2011), we assume that Chinese university teachers' behavioral intentions toward the new promotion system can be influenced by institutional support for professional growth, nonmonetary cost-benefit and practicality appraisal of the new system, and participation in decision-making.

Furthermore, as suggested by scholars (Han, 2021; Niu and Zhang, 2022; Zhang and Liu, 2022), a promotion system that effectively and fairly evaluates the research achievements of different disciplines may encourage teachers to engage in professional development, we add lecturers' fairness appraisal of the new system as a variable that can also affect behavioral intentions. As shown in Figure 1, both participation in decision-making and institutional support for professional growth can influence the three appraisals and behavioral intentions. Meanwhile, the three appraisals can also influence behavioral intentions.

## Methods

This descriptive, cross-sectional study employed a self-administered survey questionnaire that was developed on www.wjx.cn. The questionnaire consisted of two parts and took about 5 min to complete. The first part invited the respondents to indicate their demographic information, including gender, age bracket, academic qualifications, title, university type, and subject area. The second part included six subscales that were validated through a two-stage procedure, namely, the development stage and the judgment stage (Lynn, 1986; Haynes et al., 1995; Beck and Gable, 2001).

In the development stage, the researchers developed the items based on the School Participant Empowerment Scale by Short and Rinehart (1992) and the teacher receptivity scale by Waugh and Godfrey (1993). As the new promotion system in most universities evaluates teachers based on their performance in the three aspects of research, teaching, and social services (Liu et al., 2020; Han, 2021; Xing and Yuan, 2021; Niu and Zhang, 2022; Zhang and Liu, 2022), the items were localized to cover these three aspects of professional growth. In the judgment stage, three professors specializing in teacher development were consulted to ensure that the items were representative of the subscales and establish the content validity. Then a pilot study was conducted by distributing the link to two WeChat (a popular Chinese social media app) groups of Chinese university teachers from 6 to 9 January 2022, and 60 valid responses were collected. The majority of the pilot sample was females (73.3%), master's degree holders (65.0%), and lectureship holders (60.0%) in the subject area of arts (e.g., foreign language teaching, Chinese language and literature, education, and history) (76.7%) affiliated with ordinary public universities (65.0%). The Cronbach's α values of the six subscales were all above 0.8, which established the internal consistency.

**Figure 2:**
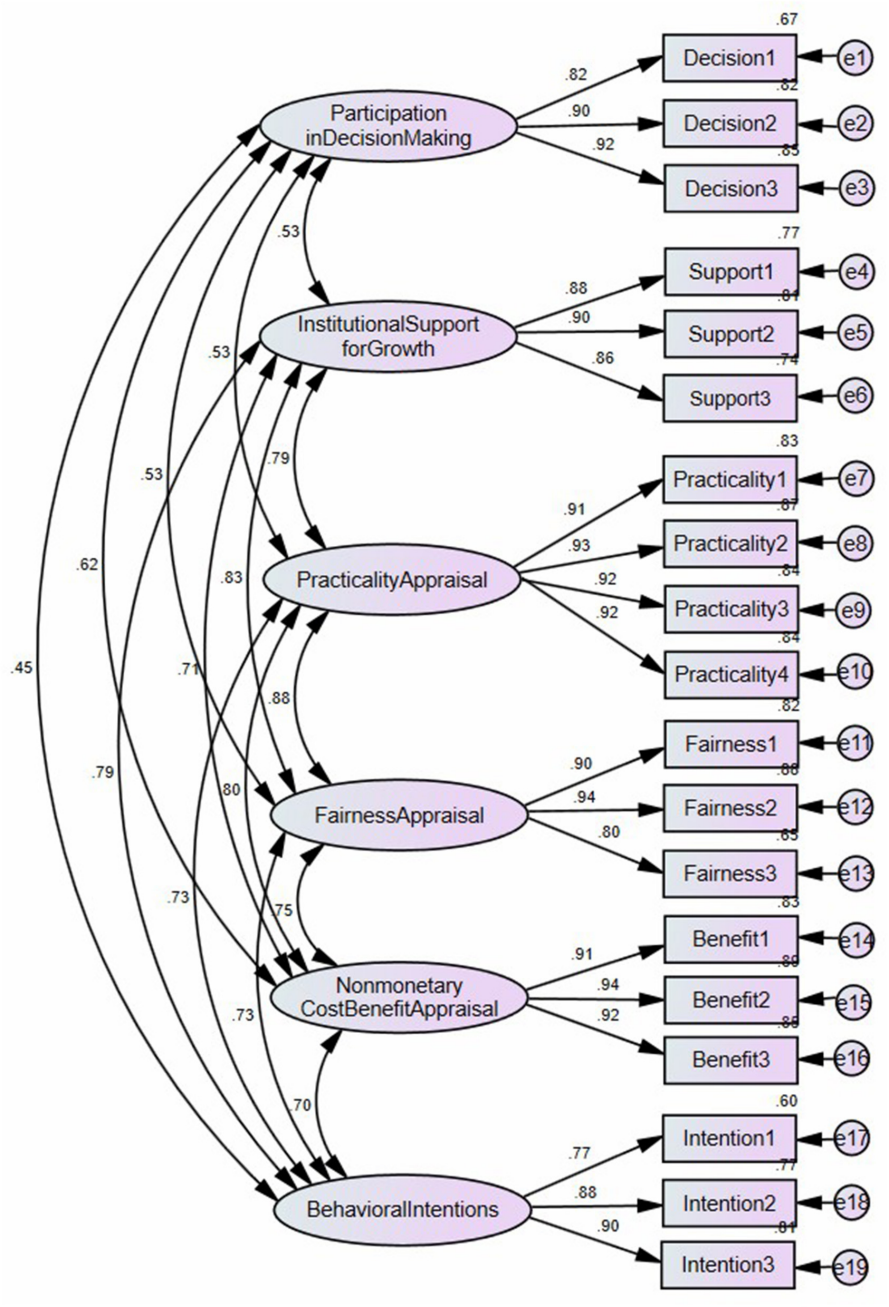
Confirmatory factor analysis (*N* = 372).

Finally, the formal survey was administered online through convenience sampling *via* WeChat, Dingtalk and Chaoxing Xuexitong (two Chinese online working platforms) from 18 to 29 April 2022. In total, 372 Chinese teachers located in northern, eastern, southern, and central China took the survey and contributed to 372 valid responses. The quantitative data of the formal survey (*N* = 372) was subject to confirmatory factor analysis (CFA) through Amos 21. As shown in Figure 2 and Table 1, CFA indicates that the 19 items load on the six intended factors and the loadings are all above 0.7, indicating unidimentionality within each subscale (Awang, 2015). Average variance extracted (AVE) values are all above 0.7, and the composite reliability (CR) values are all above 0.8, showing good convergent validity and reliability (Fornell and Larcker, 1981). Cronbach's α test and Pearson correlation coefficient test were done through SPSS 25. Cronbach's α values are all above 0.8, showing good internal consistency. Pearson's correlation coefficients are smaller than the square roots of AVE values (as shown in parentheses in Table 2), showing good discriminant validity (Fornell and Larcker, 1981). The fit indices of the six-factor model are satisfactory: χ^2^ = 378.35; df = 137; χ^2^/df = 2.76; *p* < 0.01; root mean square residual (RMR) = 0.04; standardized root mean square residual (SRMR) = 0.03; root mean square error of approximation (RMSEA) = 0.07; normed fit index (NFI) = 0.95; non-normed fit index (NNFI) = 0.96; comparative fit index (CFI) = 0.97; relative fit index (RFI) = 0.94; incremental fit index (IFI) = 0.97; Tucker Lewis index (TLI) = 0.96; goodness-of-fit index (GFI) = 0.90; adjusted goodness-of-fit index (AGFI) = 0.87; parsimony goodness-of-fit index (PGFI) = 0.65 (Browne and Cudeck, 1993; Chau and Hu, 2001; Hair et al., 2006; Bentler, 2007).

**Table 1:** Loading, AVE, CR, and Cronbach's α of the subscales (*N* = 372).

**Factor and item**	**Loading**	**AVE**	**CR**	**Cronbach's α**
**Participation in decision making**				
1. My university accepts my advice in the formulation of the new system.	0.82	0.78	0.91	0.91
2. I participate in the formulation of the new system.	0.90			
3. My advice has impact on the formulation of the new system.	0.92			
**Institutional support for professional growth**				
1. My university offers necessary support for my research development.	0.88	0.77	0.91	0.91
2. My university offers necessary support for my teaching development.	0.90			
3. My university offers necessary support for my social services development.	0.86			
**Practicality appraisal**				
1. The new system suits the positioning of my university.	0.91	0.85	0.96	0.96
2. The new system is practical for my university.	0.93			
3. The new system has evaluation rules that suit my teacher type.	0.92			
4. The new system has evaluation rules that suit my discipline.	0.92			
**Fairness appraisal**				
1. The new system strikes a balance between quantitative and qualitative evaluation.	0.90	0.78	0.91	0.91
2. The new system is fair.	0.94			
3. The new system is transparent.	0.80			
**Non-monetary cost-benefit appraisal**				
1. In weighing up the balance between the work generated for me by the new system and its potential to promote my research development, I think the new system is worthwhile.	0.91	0.85	0.95	0.95
2. In weighing up the balance between the work generated for me by the new system and its potential to promote my teaching development, I think the new system is worthwhile.	0.94			
3. In weighing up the balance between the work generated for me by the new system and its potential to promote my social services development, I think the new system is worthwhile.	0.92			
**Behavioral intentions**				
1. I will increase my research efforts according to the new system.	0.77	0.73	0.89	0.89
2. I will increase my teaching efforts according to the new system.	0.88			
3. I will increase my social services efforts according to the new system.	0.90			

**Table 2:** Discriminant validity of the subscales (*N* = 372).

**Factor**	**Participation in decision making**	**Institutional support for professional growth**	**Practicality appraisal**	**Fairness appraisal**	**Non-monetary cost-benefit appraisal**	**Behavioral intentions**
Participation in decision making	(0.88)					
Institutional support for professional growth	0.50^**^	(0.88)				
Practicality appraisal	0.51^**^	0.74^**^	(0.92)			
Fairness appraisal	0.51^**^	0.77^**^	0.82^**^	(0.88)		
Non-monetary cost-benefit appraisal	0.59^**^	0.67^**^	0.76^**^	0.70^**^	(0.92)	
Behavioral intentions	0.43^**^	0.72^**^	0.67^**^	0.67^**^	0.65^**^	(0.85)

*The square roots of AVE values are in parentheses*.

** *Correlation is significant at the 0.01 level (two-tailed)*.

As shown in Table 1, the six subscales measured teachers' participation in decision-making of the promotion system reform (three items), institutional support for professional growth (three items), teachers' practicality appraisal of the new promotion system (four items), teachers' fairness appraisal of the new system (three items), teachers' nonmonetary cost-benefit appraisal of the new system (three items), and teachers' behavioral intentions to increase research, teaching, and social services efforts according to the new system (three items). All 19 items used 5-point Likert scale (from 1 – strongly disagree to 5 – strongly agree). The six subscales were developed and administered in Chinese (see Supplementary Material).

**Figure 3:**
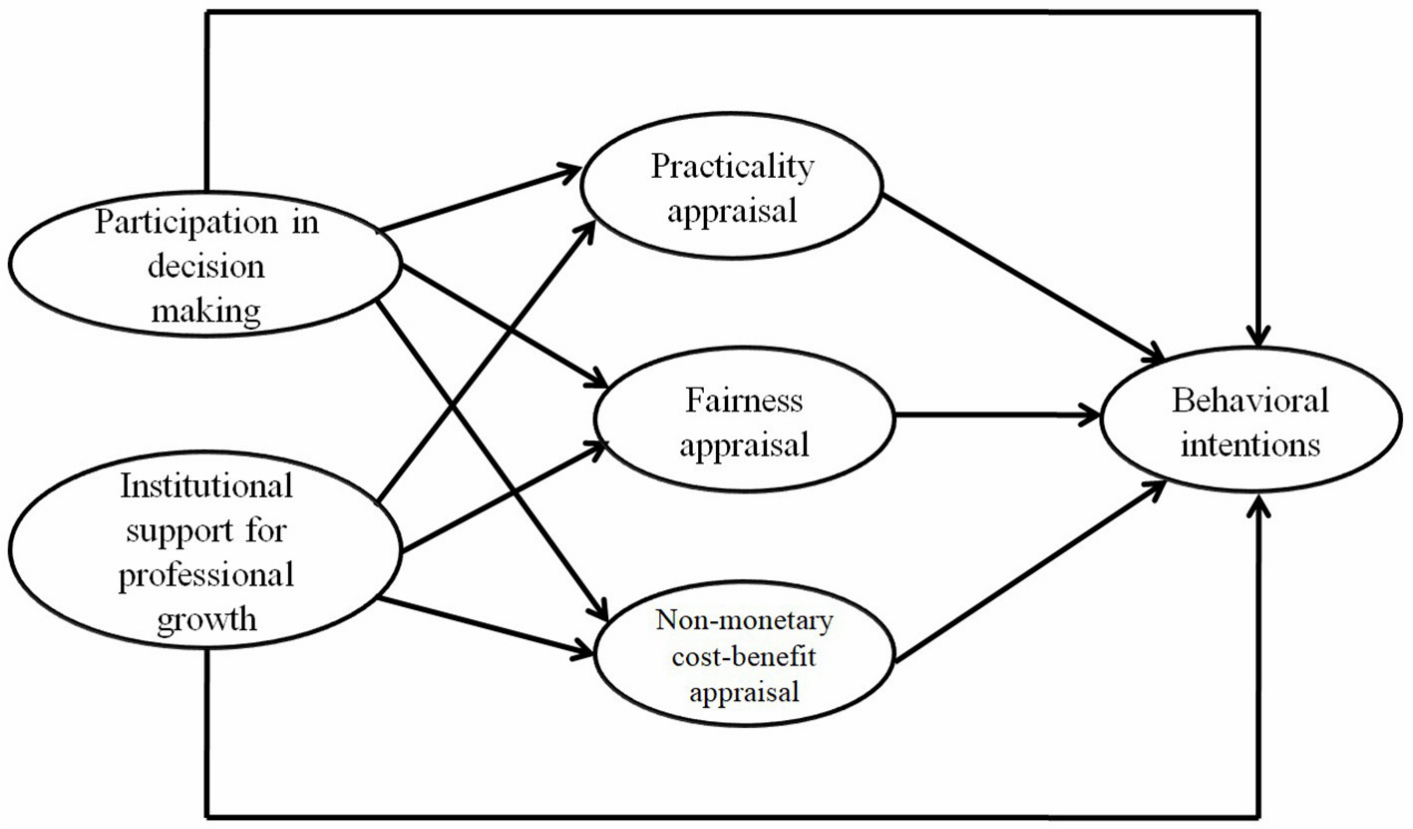
Research framework and variables.

Descriptive statistical analysis was conducted through SPSS 25 to generate means and standard deviations of the six subscales when all valid responses were included (*N* = 372) and when the 32 professors' responses were excluded (*N* = 340). Structural equation modeling (SEM) was conducted through Amos 21 to examine the direct effect of the independent variables on the dependent variables when the 32 professors' responses were taken out (*N* = 340). This exclusion was done based on the assumption that professors do not need to increase their efforts to get a promotion in the new promotion system. The research framework of the study is shown in Figure 3. Participation in decision-making and institutional support for professional growth are independent variables. Practicality appraisal, fairness appraisal, and nonmonetary cost-benefit appraisal serve as independent variables for behavioral intentions and as dependent variables for participation in decision making and institutional support for professional growth. Behavioral intentions are a dependent variable. Both participation in decision-making and institutional support for professional growth can affect the three appraisals and behavioral intentions. Meanwhile, the three appraisals can also affect behavioral intentions.

The demographic information of the formal sample of 372 Chinese university teachers is shown in Table 3. Regarding gender, there were 29.8% male and 70.2% female. Regarding age, there were 40.1% aged under 40 years, 47.3% aged 40–49 years, and 12.6% aged over 50 years. Regarding academic qualifications, there were 7.26% bachelor's degree holders, 71.77% master's degree holders, and 20.97% doctoral degree holders. Regarding title, there were 52.7% lecturers, 38.7% associate professors, and 8.6% professors. Regarding university, there were 19.6% from ordinary private universities, 73.7% from ordinary public universities, and 6.7% from high-ranking public universities, i.e., universities on the Double First-class list (China's Ministry of Education, Ministry of Finance, and National Development and Reform Commission, 2017). Regarding subject areas, there were 89.2% in arts (e.g., art, humanities, and social sciences) and 10.8% in science (e.g., natural science, computer science, medicine, and engineering).

**Table 3:** Demographic information of the 372 Chinese university teachers.

**Category**	**Frequency**	**Percent**
**Gender**		
Male	111	29.8%
Female	261	70.2%
**Age**		
≤ 39	149	40.1%
40–49	176	47.3%
≥50	47	12.6%
**Academic qualifications**		
Bachelor's degree	27	7.26%
Master's degree	267	71.77%
Doctoral degree	78	20.97%
**Title**		
Lecturer	196	52.7%
Associate professor	144	38.7%
Professor	32	8.6%
**University**		
Ordinary private	73	19.6%
Ordinary public	274	73.7%
High-ranking public	25	6.7%
**Subject area**		
Arts	332	89.2%
Science	40	10.8%

## Results

Research question 1. What are Chinese teachers' participation in decision-making and institutional support for professional growth in the promotion system reform?

Research question 2. What are Chinese teachers' practicality appraisal, fairness appraisal, and nonmonetary cost-benefit appraisal of the new promotion system?

Research question 3. What are Chinese teachers' behavioral intentions toward the new promotion system?

As shown in Table 4, the 372 teachers had low participation in decision-making (*M* = 2.09, *SD* = 0.99) and medium institutional support for professional growth (*M* = 3.09, *SD* = 0.94). In addition, they had medium practicality appraisal (*M* = 3.00, *SD* = 0.99), medium fairness appraisal (*M* = 3.12, *SD* = 0.94), and relatively low nonmonetary cost-benefit appraisal of the new system (*M* = 2.78, *SD* = 1.05). In contrast, they had relatively high behavioral intentions to increase efforts according to the new system (*M* = 3.28, *SD* = 0.90). When the 32 professors' responses were taken out, all the means became slightly lower. The results answer research questions 1–3.

**Table 4:** The teachers' profile of participation in decision-making, institutional support for professional growth, appraisal, and behavioral intentions.

**Variables**	**All responses included** **(*N* = 372)**		**Professors' responses excluded** **(*N* = 340)**	
	**Mean**	**SD**	**Mean**	**SD**
Participation in decision making	2.09	0.99	2.05	0.96
Institutional support for professional growth	3.09	0.94	3.06	0.95
Practicality appraisal	3.00	0.99	2.97	0.99
Fairness appraisal	3.12	0.94	3.10	0.93
Non-monetary cost-benefit appraisal	2.78	1.05	2.74	1.05
Behavioral intentions	3.28	0.90	3.26	0.91

**Figure 4:**
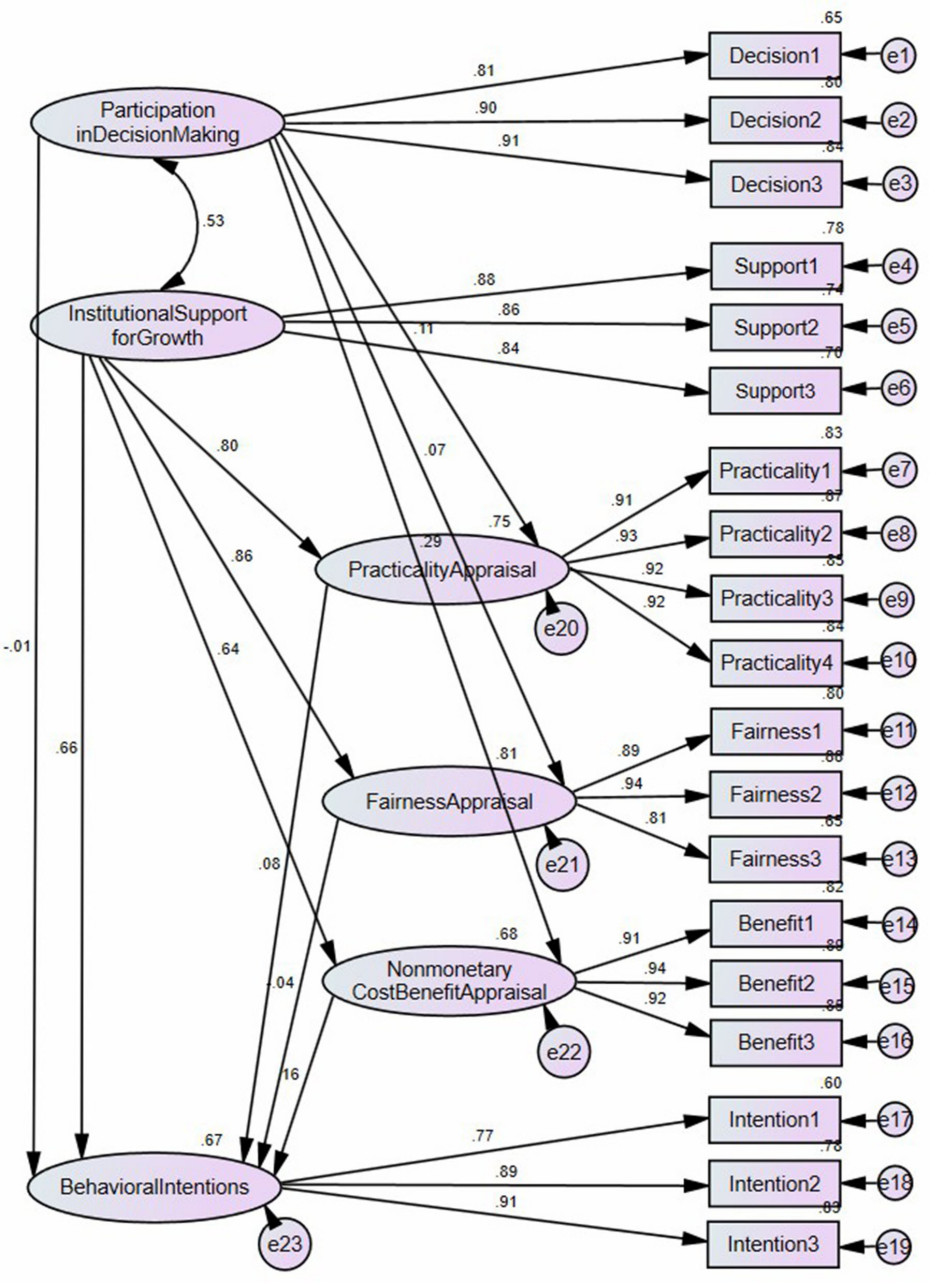
Structural equation modeling (*N* = 340). The 32 professors' responses are taken out.

The SEM of the direct effect of participation in decision-making and institutional support for professional growth on appraisal and behavioral intentions (*N* = 340) is shown in Figure 4. The fit indices of the model are satisfactory: χ^2^ = 498.43; df = 140; χ^2^/df = 3.56; *p* < 0.00; RMR = 0.05; SRMR = 0.05; RMSEA = 0.09; NFI = 0.93; NNFI = 0.94; CFI = 0.95; RFI = 0.91; IFI = 0.95; TLI = 0.94; GFI = 0.86; AGFI = 0.81; PGFI=0.63 (Browne and Cudeck, 1993; Chau and Hu, 2001; Hair et al., 2006; Bentler, 2007). The model can explain 75% of the variance in practicality appraisal, 81% of the variance in fairness appraisal, 68% of the variance in nonmonetary cost-benefit appraisal, and 67% of the variance in behavioral intentions.

Research question 4. To what extent does participation in decision-making directly affect practicality appraisal, fairness appraisal, nonmonetary cost-benefit appraisal, and behavioral intentions?

**Figure 5:**
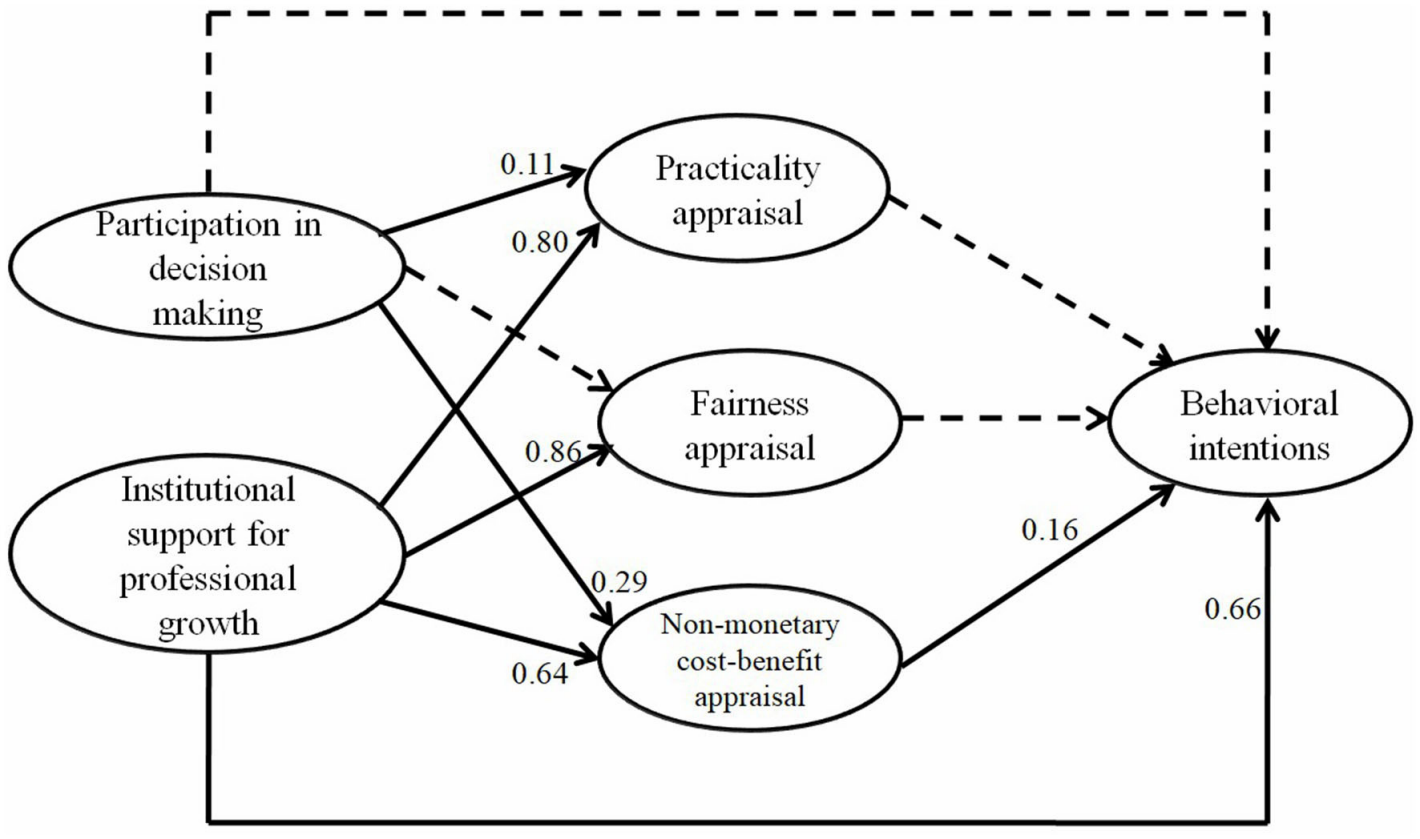
The direct effect of participation in decision-making and institutional support for professional growth on appraisal and behavioral intentions (*N* = 340). The 32 professors' responses are taken out.

As shown in Table 5 and Figure 5, participation in decision-making had a significantly and weakly direct effect on practicality appraisal (β = 0.11, *p* = 0.02) and nonmonetary cost-benefit appraisal (β = 0.29, *p* < 0.01) but no significantly direct effect on fairness appraisal (β = 0.07, *p* = 0.14) or behavioral intentions (β = −0.01, *p* = 0.91). The results answer research question 4.

**Table 5:** The direct effect of participation in decision-making and institutional support for professional growth on appraisal and behavioral intentions (*N* = 340).

**Path**			**Standardized regression weights**	**Standard error**	**Critical ratio**	***p*-value**
Practicality appraisal	←	Participation in decision making	0.11	0.05	2.41	0.02
Fairness appraisal	←	Participation in decision making	0.07	0.05	1.48	0.14
Non-monetary cost-benefit appraisal	←	Participation in decision making	0.29	0.06	5.88	0.00
Behavioral intentions	←	Participation in decision making	−0.01	0.05	−0.12	0.91
Practicality appraisal	←	Institutional support for professional growth	0.80	0.06	15.02	0.00
Fairness appraisal	←	Institutional support for professional growth	0.86	0.06	15.76	0.00
Non-monetary cost-benefit appraisal	←	Institutional support for professional growth	0.64	0.06	12.28	0.00
Behavioral intentions	←	Institutional support for professional growth	0.66	0.13	4.43	0.00
Behavioral intentions	←	Practicality appraisal	0.08	0.08	0.86	0.39
Behavioral intentions	←	Fairness appraisal	−0.04	0.10	−0.38	0.71
Behavioral intentions	←	Non-monetary cost-benefit appraisal	0.16	0.06	2.06	0.04

*The 32 professors' responses are taken out*.

Research question 5. To what extent does institutional support for professional growth directly affect practicality appraisal, fairness appraisal, nonmonetary cost-benefit appraisal, and behavioral intentions?

Institutional support for professional growth had a significantly and strongly direct effect on practicality appraisal (β = 0.80, *p* < 0.01), fairness appraisal (β = 0.86, *p* < 0.01), nonmonetary cost-benefit appraisal (β = 0.64, *p* < 0.01), and behavioral intentions (β = 0.66, *p* < 0.01). The results answer research question 5.

Research question 6. To what extent do practicality appraisal, fairness appraisal, and nonmonetary cost-benefit appraisal directly affect behavioral intentions?

Regarding the three appraisals, nonmonetary cost-benefit appraisal had a significantly and weakly direct effect on behavioral intentions (β = 0.16, *p* = 0.04), whereas practicality appraisal (β = 0.08, *p* = 0.39) and fairness appraisal (β = −0.04, *p* = 0.71) had no significantly direct effect on behavioral intentions. The results answer research question 6.

## Discussion

This study first reported that the surveyed 372 Chinese university teachers had low participation in decision-making and medium institutional support for professional growth in the promotion system reform. Meanwhile, their practicality and fairness appraisal of the new promotion system was medium and their nonmonetary cost-benefit appraisal of the new system was relatively low. Nevertheless, they had relatively high behavioral intentions to increase their engagement in research, teaching, and social services development according to the new system.

The low participation in decision-making supports the findings by Meng and Sun (2019) and Lei and Xu (2022) that Chinese university teachers perceive that they have limited impact on school-wide decisions. Additionally, in consistent with previous evidence that teachers in primary and secondary education have low actual participation in decision-making of teacher issues (Bogler, 2005; Lai and Lo, 2007; Cheng, 2008; Lee et al., 2011; Sarafidou and Chatziioannidis, 2013), this study suggests that university teachers also perceive low actual participation in decision-making of issues about teacher promotion systems and professional development. In the Chinese context, not only do teachers in primary and secondary education feel that they are expected to comply with educational policies that are often formulated in a top-down approach (Lai and Lo, 2007; Lee et al., 2011), but university teachers also perceive that they are expected to accept a new promotion system that appears to be formulated in a top-down approach. The obedient position of teachers in the promotion system reform reflects the gap between managerial power represented by policymakers and academic power represented by ordinary teachers (Romanish, 1991; Niu and Zhang, 2022).

The medium institutional support for professional growth corroborates prior evidence that teachers' perceived level of professional growth is usually moderately higher than participation in decision-making (Lee et al., 2011; Avidov-Ungar and Arviv-Elyashiv, 2018; Tindowen, 2019). Nevertheless, it still indicates that the surveyed Chinese university teachers were not satisfactory with the opportunities granted by their institutions for professional development. As stated by Han (2021), the universities in Hebei Province in China have only recently begun to give priority to teachers' continuing education and training as they carry out the performance evaluation and promotion system reforms. She suggested that universities should attach as much importance to the “dynamic” promotion of teachers' improvement as to the “static” evaluation of teachers' performance (Han, 2021, p. 86).

The medium practicality and fairness appraisal somewhat supports scholars' claim that the new promotion system is not effectively differentiating between university types, teacher types, and disciplines, and therefore it does not seem practical or fair for some university types, teacher types, and disciplines (Liu, 2018; Xing and Yuan, 2021; Fang, 2022; Niu and Zhang, 2022; Zhang and Liu, 2022). Regarding the relatively low nonmonetary cost-benefit appraisal, it can be explained by the following reasons. First of all, since the new promotion system is not effectively differentiating between university types, teacher types, and disciplines (Liu, 2018; Xing and Yuan, 2021; Fang, 2022; Niu and Zhang, 2022; Zhang and Liu, 2022), the requirements for promotion might seem overly high for some university types, teacher types, and disciplines. The individual universities, meanwhile, had not provided sufficient support for teachers to grow and meet the requirements. In weighing up the balance between the work generated for the teachers by the new system and its potential to promote their professional development, the teachers might not think it is cost-beneficial to embrace the system. In addition, the new promotion system in some universities attaches much more weight to research achievements than teaching or social services performance (Xing and Yuan, 2021; Zhang and Liu, 2022). When faced with competing demands from research and teaching, most teachers tend to choose to put more energy into research in order to meet research requirements, which will seriously affect the quality of teaching (Lai et al., 2014). Therefore, the teachers might not think the new system would benefit their teaching or social services.

Despite the unsatisfying level of participation in decision-making, institutional support for professional growth and practicality, fairness, and nonmonetary cost-benefit appraisal of the new system, the participants expressed relatively high behavioral intentions to go by the system. This contrast confirms the findings by Lee et al. (2011) and Yin et al. (2011) that, in educational reforms in China, although teachers may have relatively low participation in decision-making and institutional support, they tend to have relatively high behavioral intentions to carry out the reforms. This is partly because the Chinese culture values harmony and Chinese teachers prefer to put their energy into the improvement of their professional competencies rather than get involved in school-wide decision-making (Lo, 2005; Yin et al., 2009).

In addition to the above findings, the study also reported that, for the 340 lecturers and associate professors, participation in decision-making significantly directly affected practicality appraisal but not fairness appraisal, which to some extent supports our speculation that a low level of involvement of ordinary teachers in the formulation of the new promotion system might have resulted in some of its drawbacks, e.g., impracticality of evaluation rules for some university types, teacher types, and disciplines (Liu, 2018; Han, 2021; Xing and Yuan, 2021; Fang, 2022; Niu and Zhang, 2022; Zhang and Liu, 2022). Besides, institutional support for professional growth had a significantly and strongly direct effect on practicality appraisal and fairness appraisal. These results are somewhat consistent with Avidov-Ungar and Arviv-Elyashiv (2018) that the Israeli primary and secondary teachers with a strong sense of empowerment, which included a high perceived level of shared decision-making and professional growth, tended to consider the managerial position promotion system as fair and open.

However, practicality and fairness appraisal did not significantly directly affect behavioral intentions, which to some extent invalidates scholars' speculation that a promotion system with evaluation rules tailored to different disciplines and teachers types may facilitate teachers' endeavors in professional development for promotion (Peng, 2019; Han, 2021; Niu and Zhang, 2022; Zhang and Liu, 2022). Instead, institutional support for professional growth significantly directly affected nonmonetary cost-benefit appraisal and these two independent variables together had significantly direct influences on behavioral intentions. The results are consistent with previous findings that in the Chinese context, teachers' behavioral intentions to carry out a curricular reform are significantly and largely predicted by school support and teachers' nonmonetary cost-benefit appraisal of the new curriculum rather than teachers' participation in decision-making (Lee, 2000; Lee et al., 2011). This is, again, partly because the Chinese culture values harmony and Chinese teachers prefer to focus on improving their professional competencies rather than get involved in school-wide decision-making (Lo, 2005; Yin et al., 2009).

Nevertheless, while participation in decision-making did not have a significantly direct effect on behavioral intentions, it did have a significantly direct effect on nonmonetary cost-benefit appraisal, which in turn significantly directly affected behavioral intentions. That is to say, the teachers who considered the decision-making process as participative were more likely to recognize the nonmonetary cost-benefits of the new promotion system. In turn, these teachers were more likely to engage in development and strive for promotion. If, however, the university does not share decision-making with teachers, it may set unrealistic requirements on promotion applicants with no clue how much effort it takes for them to meet the requirements. Then teachers will find it hardly cost-beneficial to strive to satisfy the requirements and may subsequently give up their effort. Therefore, as involving teachers in the planning of professional development programs will increase the effectiveness of these programs (Tay et al., 2021), involving teachers in the formulation of new promotion systems can also promote teachers' engagement in development and pursuit of promotion in promotion system reforms.

Overall, the findings suggest that professional growth and participative decision-making are effective empowerment strategies to improve teachers' nonmonetary cost-benefit appraisal of a new promotion system and their behavioral intentions to increase efforts according to the new system. This conclusion supports scholars' claim that in promotion system reforms, policymakers should fully consider the actual conditions of the university and teachers (Xing and Yuan, 2021; Fang, 2022). If the university does not grasp its own actual conditions by consulting the faculty members, it may formulate a new promotion system that requires much more institutional support for professional growth than it can afford. When institutional support is lacking, the requirements of the new system will seem unrealistic no matter how much effort teachers put in. As a result, the reform will not be cost-beneficial for teachers and teachers may reduce their behavioral intentions to embrace the new system. Therefore, it is essential to strike a balance between the university's development goal and its reality. As stated by Cao and Lu (2007), educational reforms should be carried out in moderation and accompanied with necessary institutional support. Otherwise, teachers are likely to be overwhelmed and oppose the reform (Cao and Lu, 2007).

This study had several limitations. First, the 372 responses were collected through convenience sampling and therefore the findings cannot be generalized to all Chinese teachers. We suggest that scholars use random sampling to offer generalization of research findings. Second, the study only collected self-administered questionnaire data, which might be falsely reported by some respondents who wished to enhance their images. Hence, it is recommended that future studies employ a mixed-method research design that provides triangulation of data and a rich interpretation of contextual factors. Third, the study only reported teachers' side of the story and therefore the findings might be partial. Therefore, future studies can target both teachers and policymakers and take a comprehensive view. Finally, this study only took a snapshot of the reform at the initial stage and there is a long way to go before an effective and scientific promotion system is finalized. As the reform goes on, more factors will enter during the interplay between the university and teachers. Therefore, future studies can take a longitudinal perspective and examine how the entrance of other factors affects the current model.

## Conclusion

This study was one of the first to propose a theoretical model that integrates shared decision-making and professional growth from the teacher empowerment scale and appraisal and behavioral intentions from the teacher receptivity scale for the investigation of a promotion system reform. Localized in the Chinese context where an academic title promotion system has been ongoing since 2017, the model quantitatively examined to what extent Chinese university teachers were empowered by shared decision-making and institutional support for professional growth and how this empowerment enhanced their appraisal of and behavioral intentions toward the new promotion system. Future studies can adapt this model to the reform of teacher evaluation or promotion system in primary, secondary, and tertiary education.

The findings suggest that the surveyed 372 Chinese teachers had low participation in decision-making and medium institutional support for professional growth. Besides, they had relatively low nonmonetary cost-benefit appraisal and medium practicality and fairness appraisal of the new system. However, their behavioral intentions to increase efforts according to the new system were relatively high. In addition, participation in decision-making significantly directly affected practicality appraisal and nonmonetary cost-benefit appraisal but not fairness appraisal or behavioral intentions. Institutional support for professional growth had a significantly direct effect on practicality appraisal, fairness appraisal, nonmonetary cost-benefit appraisal, and behavioral intentions. Regarding the three appraisals, nonmonetary cost-benefit appraisal had a significantly direct effect on behavioral intentions, whereas practicality appraisal and fairness appraisal had no significantly direct effect on behavioral intentions.

The practical implications are as follows. In promotion system reforms, it is essential to strike a balance between the university's development goal and its actual conditions. The university should encourage the teachers to voice their opinions on what evaluations rules should be set and what professional development opportunities should be provided. The two empowerment strategies of participation in decision-making and professional growth can help establish a new promotion system with high nonmonetary cost-benefits for the teachers and raise their behavioral intentions to develop and pursue promotion. With these two empowerment measures adopted, both the administrators and the faculty members can work together toward the goal of promoting a better institution, faculty members, and disciplines. Additionally, the two empowerment practices can also contribute to the establishment of a new promotion system that practically and fairly evaluates each individual teacher's performance according to the characteristics of the specific university, teacher type, and discipline.

## Data Availability Statement

The original contributions presented in the study are included in the article/Supplementary Material, further inquiries can be directed to the corresponding author/s.

## Ethics Statement

Ethical review and approval was not required for the study on human participants in accordance with the local legislation and institutional requirements. Written informed consent for participation was not required for this study in accordance with the national legislation and the institutional requirements.

## Author Contributions

WP: conceptualization, methodology, data collection and analysis, and writing. SN: supervision and revision. Both authors contributed to the article and approved the submitted version.

## Conflict of Interest

The authors declare that the research was conducted in the absence of any commercial or financial relationships that could be construed as a potential conflict of interest.

## Publisher's Note

All claims expressed in this article are solely those of the authors and do not necessarily represent those of their affiliated organizations, or those of the publisher, the editors and the reviewers. Any product that may be evaluated in this article, or claim that may be made by its manufacturer, is not guaranteed or endorsed by the publisher.
