# Supplementary figures and images for: Teachers' Participation in Decision-Making, Professional Growth, Appraisal, and Behavioral Intentions in the Promotion System Reform in Chinese Universities
Source: Front Psychol. 2022 Jun 30;13:932324. doi: 10.3389/fpsyg.2022.932324 (PMC9281619; doi:10.3389/fpsyg.2022.932324)

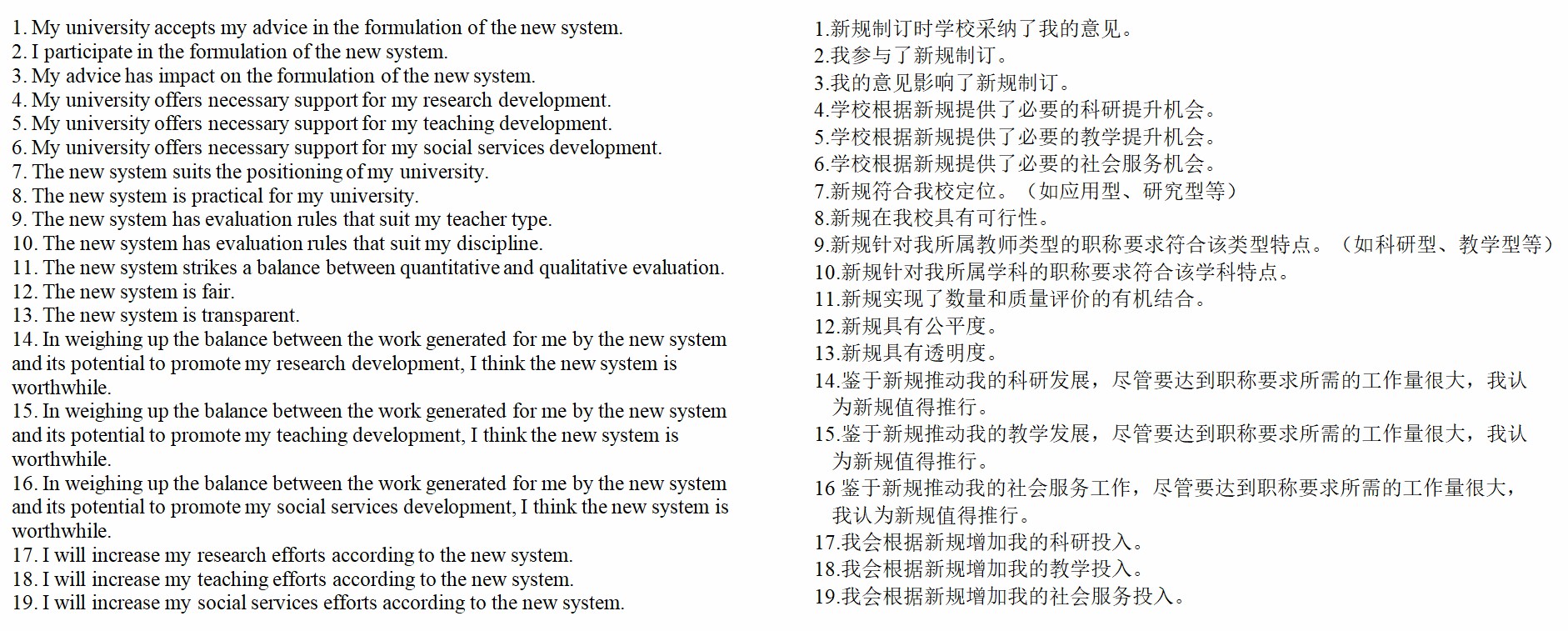

Supplement: Supplementary file 1 [file Image_1.jpg]
